# Dietary Vitamin C Intake Reduces the Risk of Type 2 Diabetes in Chinese Adults: HOMA-IR and T-AOC as Potential Mediators

**DOI:** 10.1371/journal.pone.0163571

**Published:** 2016-09-29

**Authors:** Chunling Zhou, Lixin Na, Ruiqi Shan, Yu Cheng, Ying Li, Xiaoyan Wu, Changhao Sun

**Affiliations:** 1 Department of Nutrition and Food Hygiene, the National Key Discipline, School of Public Health, Harbin Medical University, Harbin, P. R. China; 2 The Second afflicted Hospital of Harbin Medical University, Harbin, P. R. China; Johns Hopkins University Bloomberg School of Public Health, UNITED STATES

## Abstract

Despite growing interest in the protective role that dietary antioxidant vitamins may have in the development of type 2 diabetes (T2D), little epidemiological evidence is available in non-Western populations especially about the possible mediators underlying in this role. The present study aimed to investigate the association of vitamin C and vitamin E intakes with T2D risk in Chinese adults and examine the potential mediators. 178 incident T2D cases among 3483 participants in the Harbin People Health Study (HPHS), and 522 newly diagnosed T2D among 7595 participants in the Harbin Cohort Study on Diet, Nutrition and Chronic Non-communicable Diseases (HDNNCDS) were studied. In the multivariable-adjusted logistics regression model, the relative risks (RRs) were 1.00, 0.75, and 0.76 (*P*_trend_ = 0.003) across tertiles of vitamin C intake in the HDNNCDS, and this association was validated in the HPHS with RRs of 1.00, 0.47, and 0.46 (*P*_trend_ = 0.002). The RRs were 1.00, 0.72, and 0.76 (*P*_trend_ = 0.039) when T2D diagnosed by haemoglobin A_1c_ in the HDNNCDS. The mediation analysis discovered that insulin resistance (indicated by homeostasis model assessment) and oxidative stress (indicated by plasma total antioxidative capacity) partly mediated this association. But no association was evident between vitamin E intake and T2D. In conclusion, our research adds further support to the role of vitamin C intake in reducing the development of T2D in the broader population studied. The results also suggested that this association was partly mediated by inhibiting or ameliorating oxidative stress and insulin resistance.

## Introduction

Growing evidence has suggested that oxidative stress plays an important role in the pathogenesis of type 2 diabetes (T2D) by promoting insulin resistance or impairing insulin secretion [1,2,3]. Dietary antioxidative vitamins may contribute to the total antioxidant capacity of cells and plasma [4]. Thus, it seems plausible that a sufficient intake of these vitamins may play an important role in reducing the risk of T2D. Vitamin C and vitamin E, both having antioxidant properties, have been hypothesized to reduce the risk of the disease.

Some studies observed that plasma concentrations of vitamin C or vitamin E were inversely associated with the risk of T2D [5,6,7,8], which may result from increased oxidative stress related to the disease or may indicate a role of the vitamins in the development of diabetes. However, circulating concentrations of these vitamins more likely reflect the short-term intake levels before the survey [9]. Compared with circulating concentrations, dietary intakes of vitamin C or vitamin E collected with validated food frequency questionnaire (FFQ) which captured usual dietary patterns could be employed to reflect long-term levels of dietary intake. Three prospective studies and one meta-analysis have investigated the association between dietary vitamin C or vitamin E intake and incident T2D cases [10,11,12,13], but all of them were conducted in Western populations and their results were inconsistent. And to date, data regarding the mechanism of the effect of vitamin C or vitamin E intake on T2D is limited. Therefore, further research revealing the effect of dietary vitamin C or vitamin E intake on the risk of T2D in non-Western populations and exploring the potential mechanism is needed, which would provide more information for the complete elucidation of the effect of dietary antioxidants on reducing the prevalence of diabetes.

Mediation analysis was first proposed in psychological research and has been considered to be prominent statistical analysis [14]. Under appropriate causal structures justified by substantive scientific knowledge, mediation analysis addresses directly the questions of how and why the specific exposure and outcome are related by the intermediate factor through measuring its contribution to the effect of the exposure on the outcome [15,16]. Recently, mediation analysis has been paid much attention in epidemiological study; however, it has not been employed in the analysis of the association of vitamin C or vitamin E intake with T2D risk.

In the present study we aimed to add to the scientific evidence about the possible association of vitamin C and vitamin E intakes with the risk of T2D in 2 large, population-based studies conducted in urban Harbin, China. And we try to test whether there are potential mediators linking the vitamin to T2D by using mediation analysis.

## Materials and Methods

### Ethics Statement

The study protocol was approved by the Ethics Committee of the Harbin Medical University, and written informed consent was provided by all participants.

### Study population

Both the Harbin People’s Health Study (HPHS) and the Harbin Cohort Study on Diet, Nutrition and Chronic Non-communicable Diseases (HDNNCDS) are population based prospective cohort studies conducted in urban Harbin, China. The HPHS was begun first, having recruited 8940 people aged 20–74 years in 2008. Detailed in-person interviews were administered by trained personnel using a structured questionnaire to collect information on demographic characteristics, dietary habits, and lifestyles and physical condition. Previous publication has described information on the baseline methods in more detail [17]. After finishing the baseline survey, we randomly selected 4515 members (about 50.5% of total participants) to participate in the follow-up surveys due to financial limit for this study. In 2012, 4158 participants finished the first in-person follow-up survey with a response rate of 92.1%. The HDNNCDS was launched in 2010 by the national key discipline, department of nutrition and food hygiene at Harbin Medical University. Employing the same survey method used by the HPHS at baseline, we finished the baseline survey in 2012 and recruited a total of 9734 people aged 20–74 years [18]. The first follow-up survey for all cohort members in the HDNNCDS is undergoing but has not been finished yet. This study was registered at chictr.org as ChiCTR-ECH-12002721.

The present study population in the HPHS comprised of 3483 participants after excluding those who had T2D at baseline survey (*n* = 305), those who reported extreme values for total energy intake (men:> 4200 or < 800 kcal/day, women: > 3500 or < 500 kcal/day, *n* = 177), those who reported vitamin C and E supplement use (*n* = 56), and those who had missing information on education, body mass index (BMI), waist circumference, or dietary vitamin C and E intake (*n* = 137). For the HDNNCDS, the present study comprised of 7595 participants after excluding those who reported having T2D at baseline survey (*n* = 1189), those who reported extreme values for total energy intake (men:> 4200 or < 800 kcal/day, women: > 3500 or < 500 kcal/day, *n* = 368), or vitamin C and vitamin E supplement use (*n* = 89), and those who had missing information on education, BMI, waist circumference, or dietary vitamin C and vitamin E intake (*n* = 493).

### Baseline measurements and outcome ascertainment

At baseline, a FFQ was applied to collect the data regarding usual dietary intake over the past 12 months for participants in the two studies by in-person interviews. The validity and reliability of the FFQ were assessed in our previous validation study [19].The FFQ included 103 food items from 14 food groups consisting of rice, wheaten food, potato starch and its products, beans and its products, all vegetables, all fruits, livestock and its products, poultry and its products, milk and its products, eggs and its products, fish and its products, snack, beverage, and ice cream. Intakes of total energy (in kcal/day), vitamin C (in mg/day), and vitamin E (in mg/day) were estimated by the Chinese Food Composition Tables [20].

Body measurements, including height, weight, and waist circumferences, were also taken at baseline recruitment according to a standard protocol by trained interviewers at baseline survey for both cohort members. BMI (kg/m^2^) was calculated as weight (kg) divided by the square of the height in meters (m^2^). Fat mass (FM) was additionally measured using the electric impedance method with a body FM analyzer (OMRON HBF-306, Omron Corporation, Dalian, China) for participants in the HDNNCDS. Blood pressure was measured 3 times with a standard mercury sphygmomanometer on the right arm of each subject after a 10-minute rest in a sitting position, and the mean values were used for analysis for both cohort participants. Information on socio-demographic factors for both cohort members such as age, level of education (no formal education, elementary school, middle/high school, technical school/college, postgraduate degree or above), exercise regularly (any kind of recreational or sport physical activity other than walking for work or life performed three or more days per week for at least 30 minutes), current smokers (smoked at least 100 cigarettes lifetime and smoke every day or some days now), current drinkers (consumed ≥1 alcoholic drink in the 12 months before the survey), family history of diabetes (yes/no), hypertension (systolic blood pressure ≥ 140 mmHg or diastolic blood pressure ≥ 90 mmHg, and/or taking medications for hypertension), and presence of coronary heart disease (CVD) and hyperlipemia at baseline was collected by using a structured questionnaire.

An OGTT was carried out according to the World Health Organization (WHO) guidelines for both cohort members [21]. Fasting and postprandial (2 hours after drinking a 75 grams glucose-containing water) blood sample were taken from all participants at baseline. After collection, plasma samples were kept in a portable, insulated bag with ice packs (at about 0–4°C) and were processed within 6 hours for long-term storage at -80 C until analysis.

Serum insulin, plasma methane dicarboxylic aldehyde (MDA), and total antioxidative capacity (T-AOC) was measured among 1738 participants in the HPHS and 4588 participants in the HDNNCDS. These participants were about half of the participants in each studies and were randomly selected by using simple random sampling. Serum insulin was measured with an auto-analyzer using commercial kits (Centaur, Bayer Corporation, Bayer Leverkusen, Germany). Homeostasis model assessment of insulin resistance (HOMA-IR) was calculated according to the formula: Fasting glucose (mmol/L) ×Fasting insulin (mIU/L)/22.5, and HOMA-beta was calculated with the formula: 20×fasting insulin (mIU/L)/FPG (mmol/L)– 3.5[22]. MDA and T-AOC were measured with commercial kits using enzymatic methods (Jiangcheng Technology, Nanjing, China) for the above randomly selected participants in the two studies. In addition, the haemoglobin A_1c_ (HbA_1c_) assays were carried out for the previous selected 4588 participants in the HDNNCDS using high-performance liquid chromatography (HPLC) on a Bio-Rad VariantⅡVCS Hemoglobin Testing System (Shiga, Japan). The HbA_1c_ interassay and intra-assay coefficients of variation were 1.2% at a value of 5.8%, and 0.7% at a value of 8.0%.

Based on OGTT, diabetes was defined as fasting blood glucose ≥ 7.0 mmol/L, and/or 2-h glucose ≥ 11.1 mmol/L, and/or taking medications for diabetes in the follow-up data in the HPHS. While the newly diagnosed diabetes was defined as fasting blood glucose ≥ 7.0 mmol/L, and/or 2-h glucose ≥ 11.1 mmol/L in the HDNNCDS; as based on HbA_1c_, the newly diagnosed diabetes was defined as HbA_1c_ ≥ 6.5% (48mmol/mol). The OGTT was carried out in the HPHS both at baseline and in the first follow-up survey. During a median follow-up of 4.2 years for the HPHS, 183 incident cases were identified. For the HDNNCDS, 522 newly diagnosed cases were identified based on OGTT for total participants and 239 newly diagnosed cases were identified based on HbA_1c_ among the randomly selected 4588 participants. IR was defined as ≥75th percentile of the HOMA-IR among participants without T2D [23]. Participants with T2D were classified into high or low HOMA-IR groups by the median value since there is no established guideline in cut-off for HOMA-IR [24].

### Statistical analysis

Selected demographic and other factors were compared between subjects with T2D (diabetes) and without T2D (no diabetes) at baseline, using t-test for continuous variables and chi-square test for categorical variables. Vitamin C and vitamin E intakes were adjusted for total energy intake by residual method, respectively [25]. Total vitamin C or vitamin E intake (in mg/day) was categorized by tertile distribution with the lowest tertile serving as the reference, respectively. Linear associations between vitamin C or vitamin E intake and T2D were estimated per 10-mg increment of vitamin C or vitamin E intake and by analyzing linear trends across vitamin C or vitamin E intake categories using the median value of each tertile as a continuous variable. Relative risks (RRs) and their 95% confidence interval (95% CI) were estimated to measure the association of dietary vitamin C or vitamin E intake with T2D and IR by using logistic regression models. The two core models were as follows: Model 1 was adjusted for age at study recruitment (years) and sex (male/female). Model 2 was adjusted for the potential confounders including age at study recruitment (years), sex (male/female), BMI (kg/m^2^), waist circumference (cm), exercise regularly (yes/no), hypertension (yes/no), coronary heart disease (yes/no), hyperlipemia(yes/no), and total energy intake (kcal/day) in the HPHS, and additionally adjusted for body fat percentage (continuous), education (7 categories), current smokers (yes/no), and family history of T2D (yes/no) in the HDNNCDS since these variables were found to be additionally statistically significant between participants with and without T2D in the study. Multiple regression analysis was used to investigate the association of vitamin C or vitamin E intake with HbA_1c_ adjusted for the above potential confounders in the HDNNCDS.

Mediation analysis was performed to evaluate the role of HOMA-IR, HOMA-beta, MDA, or T-AOC as potential mediators of the association between the statistically significant vitamin and T2D. Mediation effect was evaluated by the degree of attenuation of the per 10-mg increment of vitamin intake effect by further adjusting for the HOMA-IR, HOMA-beta, MDA, or T-AOC in the logistic regression models. And statistical significance for the mediation effect was carried out by formally testing for the indirect effect or average causal mediation effect, using bootstrapping techniques [26,27]. All analyses were performed by using R version 3.0.3 (http://www.r-project.org/) and a two-sided *P*-value <0.05 was considered statistically significant.

## Results

Characteristics of participants with T2D (diabetes) and without T2D (no diabetes) at baseline are presented in Table 1. Compared with participants without T2D in our study, persons with T2D were significantly older, tended to have greater BMI, waist circumferences, HOMA-IR, MDA, prevalence of high hypertension, CVD, and hyperlipemia, but lower exercise, T-AOC and vitamin C intake. Persons with T2D were additionally tended to have greater body fat percentage, prevalence of family history of diabetes, to be men participants, or current smokers, but lower years of education, vitamin E intake, and HOMA-beta in the HDNNCDS, whereas these characteristics were not statistically significant in the HPHS. Persons with T2D were additionally to have lower total energy intake in the HPHS but not in the HDNNCDS. Difference in current drinking between persons with and without T2D was not statistically significant in the two studies.

**Table 1:** Baseline characteristics of participants according to type 2 diabetes status in the Harbin People’s Health Study (HPHS, 2008) and in the Harbin Cohort Study on Diet, Nutrition and Chronic Non-communicable Diseases (HDNNCDS, 2010–2012).

Variable	HPHS			HDNNCDS		
	Diabetes(*n* = 178)	No diabetes(n = 3,305)	*P* ^1^	Diabetes(*n* = 522)	No diabetes (*n* = 7,073)	*P* ^a^
Age at recruitment^b^ (years)	53.71±10.29	49.69±10.33	<0.0001	54.74±9.31	49.29±10.27	<0.0001
BMI^b^ (kg/m^2^)	26.52±4.18	24.94±3.37	<0.0001	26.09±3.62	24.75±3.47	<0.0001
Waist circumference^b^ (cm)	88.23±11.65	83.82±9.81	<0.0001	89.82±9.86	85.04±10.20	<0.0001
Body fat percentage^b^	NA^c^	NA^c^		31.23±6.11	30.31±5.72	<0.0001
Education (%)						
No formal education	3.37	1.86	0.076	3.10	1.56	<0.0001
Elementary school	9.55	5.36		8.18	4.65	
Middle school	30.90	30.12		30.66	23.39	
High school/secondary technical school	26.97	34.59		32.72	34.65	
Technical school/college	28.09	27.44		25.20	34.87	
Postgraduate degree or above	1.12	0.62		0.51	0.88	
Male (%)	35.96	31.84	0.268	43.92	34.55	<0.0001
Exercised regularly (%)	55.85	66.85	0.005	45.68	54.38	<0.0001
Current smokers (%)	19.66	17.48	0.472	20.12	17.01	0.0011
Current drinker (%)	35.39	37.92	0.5111	33.12	35.39	0.11
Family history of diabetes (%)	10.67	12.79	0.421	26.97	13.87	<0.0001
Hypertension (%)	52.00	37.02	<0.0001	59.45	35.28	<0.0001
Coronary heart disease (%)	30.90	20.07	<0.0001	31.77	16.25	<0.0001
Hyperlipemia (%)	33.33	23.23	0.003	41.15	20.96	<0.0001
Total energy intake^b^ (kcal/day)	2038.86±632.88	2146.61±641.39	0.034	2213.02±679.56	2246.55±656.97	0.09
Dietary vitamin C intake^b^ (mg/day)	90.40±60.56	106.49±70.24	0.012	92.54±60.28	102.26±67.31	<0.0001
Dietary vitamin E intake^b^ (mg/day)	11.22±4.96	11.85±4.83	0.094	11.84±4.65	12.21±4.70	0.013
HOMA-IR^b^	2.95±4.25	1.72±2.04	0.005	3.60±3.93	1.72±1.81	<0.0001
HOMA-B^b^	196.50±263.99	243.17±467.48	0.325	130.13±521.69	287.37±876.23	<0.0001
MDA^b^ (nmol/ml)	6.22±1.98	4.10±3.75	0.049	6.12±1.83	4.13±3.36	0.041
T-AOC^b^ (U/ml)	1.85±0.58	2.99±1.51	<0.0001	1.86±0.51	2.94±1.46	<0.0001

^a^ T-tests were used for continuous variables; chi-square tests were used for categorical variables.

^b^ Mean±SD(all such values).

^c^ NA, not available.

After adjustment for potential confounding factors, vitamin C intake was associated with a reduced risk of T2D (Table 2). Compared with participants in the lowest tertile, the RRs (95% CIs) for those in the second and third tertiles were 0.47(0.32–0.71) and 0.46 (0.30–0.71) (*P*_trend_ = 0.002) in the HPHS and they were 0.75(0.65–0.88) and 0.76(0.64–0.89) (*P*_trend_ = 0.003) in the HDNNCDS, respectively. In addition, as for the cases diagnosed by HbA_1c_ in the HDNNCDS, RRs (95% CIs) were 0.72(0.58–0.89) and 0.76(0.60–0.95), respectively (*P*_trend_ = 0.039), the inverse association of vitamin C intake with HbA_1c_ was observed in the HDNNCDS with *β*(standard error, SE) of -0.00128 (0.0002) (*P*<0.0001), which was independent of the potential confounders. Analyzing the association of vitamin C intake with T2D by per 10-mg increment of vitamin C intake showed comparable results after adjustment for potential confounding factors (in the HPHS: RRs (95% CIs): 0.962(0.934–0.991), *P*_trend_ = 0.0294; in the HDNNCDS: RRs (95% CIs): 0.978(0.966–0.990), *P*_trend_ = 0.0003). However, vitamin E intake was not associated with HbA_1c_ or the risk of T2D in both studies.

**Table 2:** Adjusted relative risks (RRs) (and 95% confidence interval) of type 2 diabetes diagnosed by the oral glucose tolerance test or haemoglobin A_1c_ (HbA_1c_) across tertiles of dietary vitamin C intake, the Harbin People’s Health Study (HPHS, 2008–2012) and in the Harbin Cohort Study on Diet, Nutrition and Chronic Non-communicable Diseases (HDNNCDS, 2010–2012).

	Tertile of vitamin intake (mg/day)			*P*_trend_	Per 10 mg
	Tertile1	Tertile2	Tertile3		
**HPHS(*n* = 3,483)**					
Vitamin C intake	<69.08	≥69.08–116.60	≥116.60		
No. of cases	85	48	50		
Age and sex-adjusted relative risk	1.00	0.49(0.34–0.72)	0.51(0.35–0.74)	0.002	-0.962(-0.934–0.991)
Multivariate relative risk^1^	1.00	0.47(0.32–0.71)	0.46(0.30–0.71)	0.002	-0.963(-0.931–0.996)
Vitamin E intake	<9.07	≥9.07–12.78	≥12.78		
No. of cases	78	50	55		
Age and sex-adjusted relative risk	1.00	0.60(0.41–0.88)	0.68(0.47–1.01)	0.08	-0.881(-0.648–1.199)
Multivariate relative risk^1^	1.00	0.57(0.37–0.87)	0.71(0.39–1.28)	0.37	1.143(-0.718–1.817)
**HDNNCDS**					
**Type 2 diabetes diagnosed by OGTT (*n* = 7,595)**					
Vitamin C intake	<67.56	≥67.56–109.93	≥109.93		
No. of cases	261	133	128		
Age and sex-adjusted relative risk	1.00	0.73 (0.63–0.84)	0.75(0.63–0.84)	0.0003	-0.977(-0.966–0.987)
Multivariate relative risk^2^	1.00	0.75(0.65–0.88)	0.76(0.64–0.89)	0.003	-0.978(-0.966–0.990)
Vitamin E intake	<9.67	≥9.67–13.49	≥13.49		
No. of cases					
Age and sex-adjusted relative risk	1.00	0.99(0.97–1.00)	1.057(1.051–1.064)	0.09	-0.919(-0.818–1.032)
Multivariate relative risk^2^	1.00	0.90(0.72–1.11)	0.89(0.65–1.20)	0.46	1.082(-0.870–1.345)
**Type 2 diabetes diagnosed by HbA**_**1c**_ **(*n* = 4,588)**					
Vitamin C intake	<68.19	≥68.19–112.10	≥112.10		
No. of cases	118	63	58		
Age and sex-adjusted relative risk	1.00	0.69(0.56–0.84)	0.73(0.60–0.90)	0.009	-0.974(-0.950–0.999)
Multivariate relative risk^2^	1.00	0.72(0.58–0.89)	0.76(0.60–0.95)	0.039	-0.971(-0.951–0.999)
Vitamin E intake	<9.66	≥9.66–13.49	≥13.49		
No. of cases	80	77	82		
Age and sex-adjusted relative risk	1.00	0.89(0.64–1.23)	0.91(0.65–1.26)	0.58	1.041(-0.806–1.344)
Multivariate relative risk^2^	1.00	0.84(0.56–1.25)	0.75(0.43–1.30)	0.32	1.149(-0.778–1.697)

^1^Adjusted for age at study recruitment, sex, body mass index, waist circumference, exercise regularly, total energy intake, hypertension, coronary heart disease, and hyperlipemia.

^2^Adjusted for age at study recruitment, sex, body mass index, waist circumference, exercise regularly, total energy intake, hypertension, coronary heart disease, hyperlipemia, body fat percentage, education, current smoking, and family history of type 2 diabetes.

Table 3 presents the association of vitamin C intake with IR. Vitamin C intake was associated with a decreased risk of IR in participants without T2D. Compared with the participants in the lowest tertile, the RRs (95% CIs) for those in the second and third tertiles were 0.96(0.64–1.45) and 0.88(0.69–1.02) (*P*_trend_ = 0.049) in the HPHS and the RRs (95% CIs) were 0.997(0.82–1.21) and 0.82(0.67–1.01) (*P*_trend_ = 0.043) in the HDNNCDS. Either in the HPHS or in the HDNNCDS, the participants with T2D in the second tertile of vitamin C intake have a decreased risk of having higher HOMA-IR compared with those in the lowest tertile (RRs (95% CIs) of 0.17(0.05–0.66) in the HPHS, and RRs (95%CIs of 0.84(0.58–0.95) in the HDNNCDS), however, the trends for increasing vitamin C intake with decreasing levels of HOMA-IR were not statistically significant. Additionally, vitamin E intake is not associated with IR in both studies (S1 Table).

**Table 3:** Adjusted relative risks (RRs) (and 95% confidence intervals) of insulin resistance across tertiles of dietary vitamin C intake in the Harbin People’s Health Study (HPHS, 2008–2012) and in the Harbin Cohort Study on Diet, Nutrition and Chronic Non-communicable Diseases (HDNNCDS, 2010–2012).

	Tertiles of dietary vitamin C intake (mg/day)			*P*_trend_
**HPHS**				
Diabetic participants(*n* = 117)	<57.26	≥57.26–103.18	≥103.18	
No. of cases	23	16	20	
Age and sex-adjusted relative risk	1.00	0.36(0.14–0.94)	0.52(0.20–1.35)	0.48
Multivariate relative risk^1^	1.00	0.17(0.05–0.66)	0.29(0.08–1.08)	0.37
Non-diabetic participants(*n* = 1,621)	<70.53	≥70.53–115.57	≥115.57	
No. of cases	74	91	90	
Age and sex-adjusted relative risk	1.00	0.93(0.65–1.34)	1.08(0.76–1.53)	0.56
Multivariate relative risk^1^	1.00	0.96(0.64–1.45)	0.88(0.69–1.02)	0.049
**HDNNCDS**				
Diabetic participants(*n* = 239)	<61.71	≥61.71–104.33	≥104.33	
No. of cases	40	42	41	
Age and sex-adjusted odds ratio	1.00	0.88(0.69–0.99)	0.87(0.61–1.25)	0.43
Multivariate odds ratio^2^	1.00	0.84(0.58–0.95)	0.78(0.51–1.19)	0.22
Non-diabetic participants(*n* = 4,349)	<68.42	≥68.42–111.30	≥111.30	
No. of cases	315	352	309	
Age and sex-adjusted odds ratio	1.00	1.03(0.86–1.23)	0.84(0.70–1.00)	0.034
Multivariate odds ratio^2^	1.00	0.997(0.82–1.21)	0.82(0.67–1.01)	0.043

^1^Adjusted for age at study recruitment, sex, body mass index, waist circumference, exercise regularly, total energy intake, hypertension, coronary heart disease, and hyperlipemia.

^2^Adjusted for age at study recruitment, sex, body mass index, waist circumference, exercise regularly, total energy intake, hypertension, coronary heart disease, hyperlipemia, body fat percentage, education, current smoking, and family history of type 2 diabetes.

No significant interaction between any of the mediators and vitamin C intake was observed in the present study, and thus, models assuming no interaction between exposure and mediators were adopted. The results of mediation analysis are shown in Table 4. In mediation assessment, 178 incident cases of T2D among 3483 participants in the HPHS with other information on vitamin C and mediators at baseline were included in the analysis. We observed statistically significant indirect effects of HOMA-IR and T-AOC (in the HPHS: -0.00018, 95% bias corrected intervals: -0.00031, -0.000035, *P* = 0.03 for HOMA-IR, and -0.00020, 95% bias corrected intervals: -0.00038, -0.000053, *P* = 0.04 for T-AOC; in the HDNNCDS: -0.00020, 95% bias corrected intervals: -0.00038, -0.000033, *P* = 0.02 for HOMA-IR, and -0.00022, 95% bias corrected intervals: -0.00040, -0.000034, *P* = 0.03 for T-AOC), suggesting that the association between vitamin C intake and T2D was potentially mediated by HOMA-IR and T-AOC. Although positive association of vitamin C intake with HOMA-beta was observed in the HDNNCDS with *β*(SE) of 0.43 (0.22) (*P* = 0.046), no statistically significant mediation effect was found for HOMA-beta (data not shown).

**Table 4:** Causal associations between per 10-mg increment of dietary vitamin C intake and type 2 diabetes in the Harbin People’s Health Study (HPHS, 2008–2012) and in the Harbin Cohort Study on Diet, Nutrition and Chronic Non-communicable Diseases (HDNNCDS, 2010–2012).

	Mediator	Indirect effect (95% bias ias corrected interval)	*P*-value
HPHS^1^			
	HOMA-IR	-0.00018(-0.00031,-0.000035)	0.03
	T-AOC	-0.00020(-0.00038,-0.000053)	0.04
	MDA	-0.00053(-0.0017,0.00046)	0.34
HDNNCDS^2^			
	HOMA-IR	-0.00020(-0.00038,-0.000033)	0.02
	T-AOC	-0.00022(-0.00040,-0.000034)	0.03
	MDA	-0.00053(-0.00017,0.00045)	0.21

^1^Adjusted for age at study recruitment, sex, body mass index, waist circumference, exercise regularly, total energy intake, hypertension, coronary heart disease, and hyperlipemia.

^2^Adjusted for age at study recruitment, sex, body mass index, waist circumference, exercise regularly, total energy intake, hypertension, coronary heart disease, hyperlipemia, body fat percentage, education, current smoking, and family history of type 2 diabetes.

We estimated the sex-specific diabetes risk probability affected by different vitamin C intake levels with otherwise the following adjusted characteristics assumed (mean values for continuous variables, one category for categorical variables) in logistics regression models: age at study recruitment (49.76 years for men and 50.30 years for women), BMI (25.94 kg/m^2^ for men and 24.71 kg/m^2^ for women), WC (90.07 cm for men and 81.56 cm for women), total energy intake (2362.83 kcal/day for men and 2026.14 kcal/day for women), exercise regularly, no hypertension, no coronary heart disease, and no hyperlipemia; in the HDNNCDS: age at study recruitment (49.28 years for men and 49.60 years for women), BMI (25.63 kg/m^2^ for men and 24.46 kg/m^2^ for women), WC (90.76 cm for men and 82.56 cm for women), body fat percentage (26.73 for men and 32.45 for women), total energy intake (2550.74 kcal/day for men and 2198.71 kcal/day for women), exercise regularly, no hypertension, no coronary heart disease, no hyperlipemia, and no family history of T2D. As shown in Table 5, the estimated diabetes probabilities are less than 5% when vitamin C intakes are at higher levels (in the HPHS: > 100 mg/day for men and > 120 mg/day for women, in the HDNNCDS: > 140 mg/day for men and > 100 mg/day for women).

**Table 5:** Estimated diabetes probability by categories of vitamin C intake after adjusted for potential confounders with assumed values in logistics regression models in the Harbin People’s Health Study (HPHS, 2008–2012) and in the Harbin Cohort Study on Diet, Nutrition and Chronic Non-communicable Diseases (HDNNCDS, 2010–2012).

Categories of vitamin C intake(mg/day)	HPHS				HDNNCDS			
	Men(*n* = 1252)		Women(*n* = 2231)		Men(*n* = 2610)		Women(*n* = 4985)	
	*n*	Estimated probability (%)	*n*	Estimated probability (%)	*n*	Estimated probability (%)	*n*	Estimated probability (%)
<40	138	6.79	239	6.88	412	6.83	471	6.36
≥40–60	161	6.32	270	6.42	439	6.47	683	6.00
≥60–80	155	5.89	275	5.96	478	6.11	780	5.64
≥80–100	167	5.42	306	5.50	387	5.75	789	5.28
≥100–120	137	4.96	265	5.04	281	5.39	672	4.92
≥120–140	111	4.50	242	4.58	197	5.03	363	4.56
≥140–160	132	4.04	207	4.12	108	4.67	315	4.20
≥160–180	89	3.58	145	3.66	75	4.31	235	3.84
≥180	162	3.11	282	3.20	233	3.95	677	3.48

## Discussion

In these two studies of Chinese adults, we observed that vitamin C intake was significantly associated with a reduced risk of T2D at baseline in the HDNNCDS, and this association was further validated in the follow-up surveys in the HPHS. The estimated diabetes probability would be less than 5% when intake level of vitamin C was larger than 140 mg/day. It is noteworthy that we found that the effect of vitamin C intake on T2D may be mediated by inhibiting or ameliorating oxidative stress (indicated by T-AOC) and insulin resistance (indicated by HOMA-IR) by using mediation analysis.

Feskens et al observed that vitamin C intake was significantly lower among incident cases of T2D among men participants in the Dutch and Finnish cohorts of the Seven Countries Study [11]. Montonen et al did not find significant association between dietary vitamin C intake and incident T2D in the Finnish Mobile Clinic Health Examination Survey, but the incident cases were ascertained by a nationwide registry of patients receiving drug reimbursement not by glucose levels [10]. The inverse association of vitamin C intake with the risk of T2D in the present study is in agreement with those found by Feskens et al, in which cases with T2D were diagnosed by OGTT as well. Our results adds further support to previous findings that vitamin C intake may has a protective effect against the development of diabetes. In addition, we would suggest increasing the intake level of vitamin C to be at least 140 mg/day if possible in order to decrease the diabetes probability to be less than 5% based on the present results. The relationship between T2D and vitamin E intake in previous studies is inconsistent as well. Vitamin E intake has been associated with a reduced risk of T2D in the Finnish Mobile Clinic Health Examination Survey [10], while the mean intake of vitamin E did not differ between nondiabetic and diabetic people in the Insulin Resistance and Atherosclerosis Study [12]. In this study, we found no statistically significant association of T2D with vitamin E intake.

As known, vitamin C is an electron donor and its known physiological and biochemical actions are all due to this function. By donating its electrons, vitamin C can prevent other compounds from being oxidized and so is called an antioxidant. A free radical, ascorbyl radical or semidehydroascorbic acid, will be formed after the loss of one electron of vitamin C during its reaction. It is noteworthy that ascorbyl radical is relatively stable with a half-life of 10^−5^ seconds and is fairly unreactive as compared with other free radicals, which makes it be a preferred antioxidant [28]. However, data, examining whether vitamin C protecting against T2D risk is through its antioxidant activity, is limited. To clarify this speculation, we performed mediation analysis in the present study. Our data showed that the association between vitamin C intake and T2D was mediated by oxidative stress as determined by plasma T-AOC levels, which adds further support to previous findings that vitamin C intake reduces the risk of developing T2D possibly by inhibiting or ameliorating oxidant damage through its antioxidative function.

IR is a main pathophysiological component in T2D [29,30], and the positive prevention of IR plays an important role in controlling T2D. In the present study, dietary vitamin C intake appeared to decrease the risk of insulin resistance (indicated by HOMA-IR) in nondiabetic participants, and it also decreased the risk of having higher HOMA-IR among diabetic participants who were in the second tertile compared to those in the lowest tertile. Consequently, we would hypothesize that the favorable protective effect of vitamin C intake on the risk of T2D might be possibly explained by its effect on IR. To verify this speculation, mediation analysis was conducted in this study. When HOMA-IR was further included in the logistic regression models of vitamin C intake and T2D, HOMA-IR, but not vitamin C intake, remained significantly associated with T2D, suggesting that insulin resistance may be mechanistically involved as a mediator in the association between vitamin C intake and T2D.

In addition, we employed HbA_1c_ which captures the degree of glucose exposure overtime to diagnose T2D [31,32,33], and the results were concordance with those from OGTT. Compared with OGTT, the application of HbA_1c_ is of greater convenience (fasting not required) and less day-to-day perturbations during stress and illness [34]. Although the regression coefficient was relatively small, a statistically significant inverse association was observed between vitamin C intake and HbA_1c_ after adjustment for potential confounders. It has been suggested by Davie et al that the effect of vitamin C intake on protein glycation was a competition of ascorbic acid and dehydroascorbic acid with glucose for reaction with the protein amino group on the hemoglobin beta chain, thereby inhibiting glycation [35]. Nevertheless, the association between vitamin C intake and HbA_1c_ was evaluated in the HDNNCDS at baseline, which needs further validation in follow-up surveys.

Our study has several significant strengths: both of the studies were population-based and the follow-up surveys were conducted for the HPHS with a high follow-up rate. The T2D cases were diagnosed by OGTT and/or HbA_1c_, the results were validated in two studies, and a wide range of potential confounders were adjusted for in the two studies. Specifically, we conducted the mediation analysis to explore the underlying mechanism of the effect of vitamin C intake on T2D. However, some limitations of this study must be considered. Although we adjusted for confounders, we cannot exclude the possibility of residual confounding. And there is the possibility that the association between vitamin C intake and T2D might be explained by other confounders at the same time, such as other components of vitamin C-containing foods. In addition, the number of participants who took vitamin C and vitamin E supplementation was relatively small and they were excluded in the present study, which prevented us from further analyzing the effect of vitamin C and vitamin E intake derived from supplement on T2D.

In summary, results from the present study suggest that dietary vitamin C intake plays a protective role in the development of T2D. The risk of developing diabetes probability would be less than 5% when the intake level of vitamin C was larger than 140 mg/day. And this association may be mediated by inhibiting or ameliorating oxidative stress and insulin resistance. The findings in the present study add to the limited data available on association of vitamin C intake with the risk of T2D in non-Western populations.

## Supporting Information

S1 Table S1_Table (DOCX). Adjusted relative risks (RRs)/odds ratios (ORs) (and 95% confidence intervals) of insulin resistance according to tertiles of dietary vitamin E intake in the Harbin People’s Health Study (HPHS, 2008–2012) and the Harbin Cohort Study on Diet, Nutrition and Chronic Non-communicable Diseases (HDNNCDS, 2010–2012).
(DOCX)
