# Supplementary material for: Dietary Vitamin C Intake Reduces the Risk of Type 2 Diabetes in Chinese Adults: HOMA-IR and T-AOC as Potential Mediators
Source: PLoS One. 2016 Sep 29;11(9):e0163571. doi: 10.1371/journal.pone.0163571 (PMC5042374; doi:10.1371/journal.pone.0163571)
Supplement: S1 Table — Adjusted relative risks (RRs)/odds ratios (ORs) (and 95% confidence intervals) of insulin resistance according to tertiles of dietary vitamin E intake in the Harbin People’s Health Study (HPHS, 2008–2012) and the Harbin Cohort Study on Diet, Nutrition and Chronic Non-communicable Diseases (HDNNCDS, 2010–2012). (DOCX) [file pone.0163571.s001.docx]

**S1 Table.** Adjusted relative risks (RRs)/odds ratios (ORs) (and 95% confidence intervals) of insulin resistance according to tertiles of dietary vitamin E intake in the Harbin People’s Health Study (HPHS, 2008-2012) and the Harbin Cohort Study on Diet, Nutrition and Chronic Non-communicable Diseases (HDNNCDS, 2010-2012)

|  | Tertiles of dietary vitamin E intake (mg/day) | | | *P*-trend |
| --- | --- | --- | --- | --- |
| **HPHS** |  |  |  |  |
| Diabetic participants(*n*=117) | <8.62 | ≥8.62-11.27 | ≥11.27 |  |
| No. of cases | 17 | 18 | 17 |  |
| Age and sex-adjusted relative risk | 1.00 | 0.91(0.35-2.38) | 0.96(0.37-2.50) | 0.97 |
| Multivariate relative risk^1^ | 1.00 | 0.37(0.11-1.26) | 0.22(0.04-1.37) | 0.12 |
| Non-diabetic participants(*n*=1,621) | <9.27 | ≥9.27-12.96 | ≥12.96 |  |
| No. of cases | 69 | 75 | 76 |  |
| Age and sex-adjusted relative risk | 1.00 | 1.12(0.77-1.63) | 1.09(0.75-1.59) | 0.70 |
| Multivariate relative risk^1^ | 1.00 | 0.98(0.63-1.53) | 0.71(0.38-1.35) | 0.27 |
| **HDNNCDS** |  |  |  |  |
| Diabetic participants(*n*=239) | <9.38 | ≥9.38-13.23 | ≥13.23 |  |
| No. of cases | 41 | 37 | 45 |  |
| Age and sex-adjusted odds ratio | 1.00 | 0.62(0.35-1.12) | 0.84(0.47-1.50) | 0.72 |
| Multivariate odds ratio^2^ | 1.00 | 0.41(0.20-0.83) | 0.36(0.13-1.00) | 0.06 |
| Non-diabetic participants(*n*=4,349) | <9.72 | ≥9.72-13.53 | ≥13.53 |  |
| No. of cases | 329 | 324 | 323 |  |
| Age and sex-adjusted odds ratio | 1.00 | 0.98(0.80-1.17) | 0.95(0.79-1.14) | 0.59 |
| Multivariate odds ratio^2^ | 1.00 | 0.91(0.72-1.14) | 0.79(0.57-1.08) | 0.14 |

^1^Adjusted for age at study recruitment, sex, body mass index, waist circumference, exercise regularly, total energy intake, hypertension, coronary heart disease, and hyperlipemia.

^2^Adjusted for age at study recruitment, sex, body mass index, waist circumference, exercise regularly, total energy intake, hypertension, coronary heart disease, hyperlipemia, body fat percentage, education, current smoking, and family history of type 2 diabetes.
